# Antitumor Activity of a Novel Tyrosine Kinase Inhibitor AIU2001 Due to Abrogation of the DNA Damage Repair in Non-Small Cell Lung Cancer Cells

**DOI:** 10.3390/ijms20194728

**Published:** 2019-09-24

**Authors:** Hwani Ryu, Hyun-Kyung Choi, Hyo Jeong Kim, Ah-Young Kim, Jie-Young Song, Sang-Gu Hwang, Jae-Sung Kim, Da-Un Kim, Eun-Ho Kim, Joon Kim, Jiyeon Ahn

**Affiliations:** 1 Division of Radiation Biomedical Research, Korea Institute of Radiological & Medical Sciences (KIRAMS), Seoul 01812, Korea; hwanya85@kirams.re.kr (H.R.); hjkim@kirams.re.kr (H.J.K.); tenderlady0513@gmail.com (A.-Y.K.); immu@kirams.re.kr (J.-Y.S.); sgh63@kirams.re.kr (S.-G.H.); jaesung@kirams.re.kr (J.-S.K.); eh140149@kirams.re.kr (E.-H.K.); 2 Department of Biology, Korea University, Seoul 02841, Korea; joonkim@korea.ac.kr; 3 Department of Medicinal Chemistry, Jungwon University, Goesan 28024, Korea; hkchoi45@jwu.ac.kr (H.-K.C.); kdw-101010@naver.com (D.-U.K.)

**Keywords:** FLT3 inhibitor, Class III RTK, NSCLC, apoptosis, cell cycle arrest, DNA damage repair, PARP-1 inhibitor

## Abstract

Class III receptor tyrosine kinase (RTK) inhibitors targeting mainly FLT3 or c-KIT have not been well studied in lung cancer. To identify a small molecule potentially targeting class III RTK, we synthesized novel small molecule compounds and identified 5-(4-bromophenyl)-N-(naphthalen-1-yl) oxazol-2-amine (AIU2001) as a novel class III RKT inhibitor. In an in vitro kinase profiling assay, AIU2001 inhibited the activities of FLT3, mutated FLT3, FLT4, and c-KIT of class III RTK, and the proliferation of NSCLC cells in vitro and in vivo. AIU2001 induced DNA damage, reactive oxygen species (ROS) generation, and cell cycle arrest in the G2/M phase. Furthermore, AIU2001 suppressed the DNA damage repair genes, resulting in the ‘BRCAness’/‘DNA-PKness’ phenotype. The mRNA expression level of *STAT5* was downregulated by AIU2001 treatment and knockdown of *STAT5* inhibited the DNA repair genes. Our results show that compared to either drug alone, the combination of AIU2001 with a poly (ADP-ribose) polymerase (PARP) inhibitor olaparib or irradiation showed synergistic efficacy in H1299 and A549 cells. Hence, our findings demonstrate that AIU2001 is a candidate therapeutic agent for NSCLC and combination therapies with AIU2001 and a PARP inhibitor or radiotherapy may be used to increase the therapeutic efficacy of AIU2001 due to inhibition of DNA damage repair.

## 1. Introduction

Lung cancer is one of the most common cancers in the world. Non-small cell lung cancer (NSCLC) accounts for more than 80% of all lung cancers, whereas small cell lung cancer represents 15–20% cases [1]. Despite advancements in our understanding of the molecular/genetic basis of lung cancer and improvements in therapy, the five-year survival rate (18%) of patients with lung cancer is lower than those with many other types of cancer, such as melanoma of the skin (92%), female breast cancer (90%), and prostate cancer (99%) in the United States [2]. Currently, platinum-based regimens are the first-line standard chemotherapy for treating NSCLC, and the second-line therapies include docetaxel, pemetrexed, or erlotinib. However, many patients with NSCLC receive third-line therapies because of no response or resistance to those therapies [3]. Therefore, development of novel drugs or strategies involving combination therapy with the existing drugs is urgently required.

The human genome encodes 58 receptor type protein tyrosine kinases (RTKs), which have been structurally classified into 20 different subfamilies, including class III RTKs. A prototype RTK has an extracellular ligand-binding domain, a single transmembrane helix, and an intracellular tyrosine kinase domain. Dysregulated RTK signaling has been implicated in the development of many human diseases. RTKs are often mutated, aberrantly overexpressed, or excessively activated in various types of cancer, and their signaling cascades affect tumor initiation, progression, malignancy, and metastasis [4]. The class III RTK family includes platelet-derived growth factor receptor (PDGFR)-α/β, c-KIT, colony stimulating factor 1 receptor (CSF1R), and FMS-like tyrosine kinase 3 (FLT-3), which play critical roles in the proliferation, differentiation, angiogenesis, and malignancy of various types of human cancers [5]. Numerous RTK inhibitors have been developed to induce cancer cell death in various tumor types.

Drugs targeting class III and class IV RTK for retarding tumor growth are attractive, as they exert their antitumor activity by regulating multiple molecular signaling mechanisms and offer the benefits of combination therapy with other therapeutic agents such as platinum-based drugs in various tumor types [6]. However, small molecule compounds inhibiting class III RTK have not been well-studied in NSCLC. Only sorafenib and sunitinib, which are multi-kinase inhibitors targeting vascular endothelial growth factor receptor (VEGFR)-2 (class IV RTK), PDGFR-β, c-KIT, and FLT3, have shown significant antitumor activity and anti-angiogenesis in the preclinical models of various tumor types, including NSCLC [7,8]. Multi-kinase inhibitors targeting mainly FLT3 are generally accepted for acute myeloid leukemia (AML) treatment but are not clinically used on solid tumors due to low efficacy. Currently, combination treatments, instead of single therapeutic agents, are being extensively investigated for the treatment of solid tumors [9,10,11,12,13].

Recently, Maifrede et al. demonstrated that the mutated FLT3-ITD augmented reactive oxygen species (ROS) levels, which induced DNA damage, resulting in mutations and chromosomal instability, and inhibition of FLT3-ITD activity by a FTL3 inhibitor AC220 (quizartinib) inhibited two major DNA double-strand break (DSB) repair pathways due to inhibiting activities of the DNA repair proteins BRCA1, BRCA2, PALB2, RAD51, and LIG4 [14]. The drug-mediated “BRCAness/DNA-PKness” phenotype can provide an opportunity to induce synthetic lethality with a poly (ADP-ribose) polymerase (PARP) inhibitor in combination therapy.

In our attempts to identify a small molecule potentially targeting the class III RTK, we designed, synthesized, and evaluated a novel multi-kinase inhibitor, AIU2001. This study aimed to investigate the antitumor effect of AIU2001 and the potential for combination therapy with AIU2001 and a PARP inhibitor or radiotherapy in NSCLC cells

## 2. Results

### 2.1. AIU2001 Inhibited the Proliferation of Human NSCLC Cells

**Figure 1:**
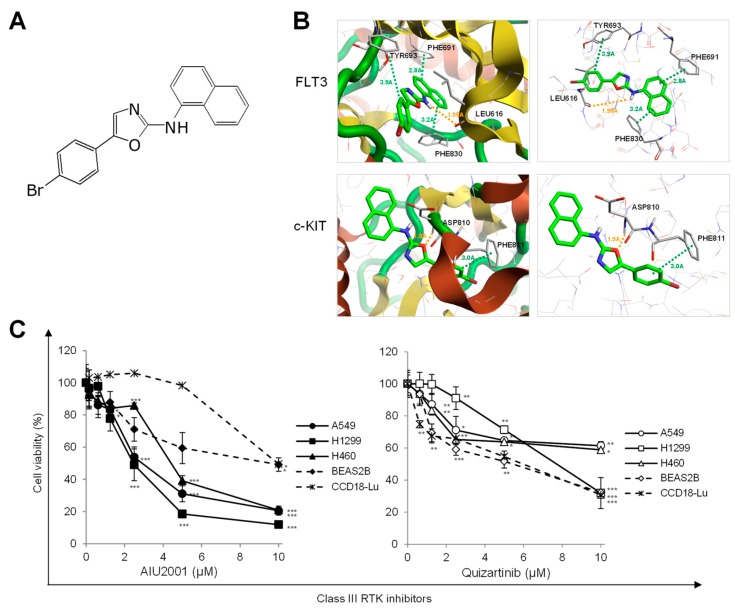
AIU2001 inhibited cell proliferation of human NSCLC cells. (**A**) Chemical structure of AIU2001. (**B**) 3D binding modes and interactions of AIU2001 withFLT3 or c-KIT were predicted using the Cresset Flare software. AIU2001 is highlighted in green. Values of the relevant distances are shown in Å. (**C**) Human NSCLC cells (A549, H1299, and H460) and human normal like lung cells (BEAS2B and CCD18-Lu) were treated with AIU2001 or quizartinib at the indicated concentrations for five days and viable cells were evaluated using the 3-(4,5-dimethylthiazol-2-yl)-2,5- diphenyltetrazolium bromide (MTT) assay. * *p* < 0.05, ** *p* < 0.01, *** *p* < 0.001 versus DMSO-treated control.

In-house RTK inhibitors were designed and synthesized based on published kinase inhibitor oxazole derivatives [15]. Among the synthesized compounds, we identified a novel class III RTK inhibitor, 5-(4-bromophenyl)-N-(naphthalen-1-yl) oxazol-2-amine, named AIU2001, which significantly inhibited the activities of class III RTK (Figure 1A and Supplementary Scheme S1). The selectivity and activity of AIU2001 was evaluated against 53 kinases covering the major oncogenic kinases of the human protein kinome (Supplementary Table S1). The in vitro kinase inhibition profile revealed that 10 µM AIU2001 selectively and potently inhibited class III RTKs such as FLT3, mutated FLT3-ITD, mutated FLT3-D865Y, FLT4 (VEGFR3), c-Kit, and mutated c-KIT-V560G, with a cut-off of ≥ 75% inhibition of activity, which was used to determine each IC50 value (Table 1).

A docking study between AIU2001 and FLT3 (PDB code: 4XUF) or c-KIT (PDB code: 6GQJ) was performed using the X-ray structures [16,17]. Pi interactions were identified between the benzyl group of AIU2001 and the Tyr693 of FLT3, and the naphthyl group of AIU2001 and both Phe691 and Phe830. In addition, a hydrogen bond was formed between the amine group of AIU2001 and the Leu616 of FLT3. Two interactions were confirmed in the complex formed between AIU2001 and c-KIT; the hydrogen bond between the oxygen of oxazole ring of AIU2001 and the Asp810 of c-KIT, and a putative pi interaction between the bromophenyl group of AIU2001 and Phe811 (Figure 1B). Therefore, AIU2001 was predicted to be capable of inhibiting FLT3 and c-KIT via direct binding to both FLT3 and c-KIT.

We observed that AIU2001 inhibited the growth of human NSCLC A549, H1299, and H460 cells and human colorectal cancer HCT116 p53^+/+^ and HCT116 p53^-/-^ cells, as well as human FLT(ITD)-positive AML MV4-11 and Molm-13 cells (Figure 1C and Supplementary Figure S1A–C). As shown in Figure 1C, AIU2001 significantly inhibited the growth of NSCLC cells, whereas growth inhibition of normal human bronchial epithelial BEAS2B cells and the lung fibroblast CCD18-Lu cells was relatively less. The growth-inhibitory effect of quizartinib (a FLT3 inhibitor and a class III RTK inhibitor) on NSCLC and normal cell lines was determined using the MTT assay. Results showed that quizartinib slightly inhibited the proliferation of NSCLC and normal cells. 

**Figure 2:**
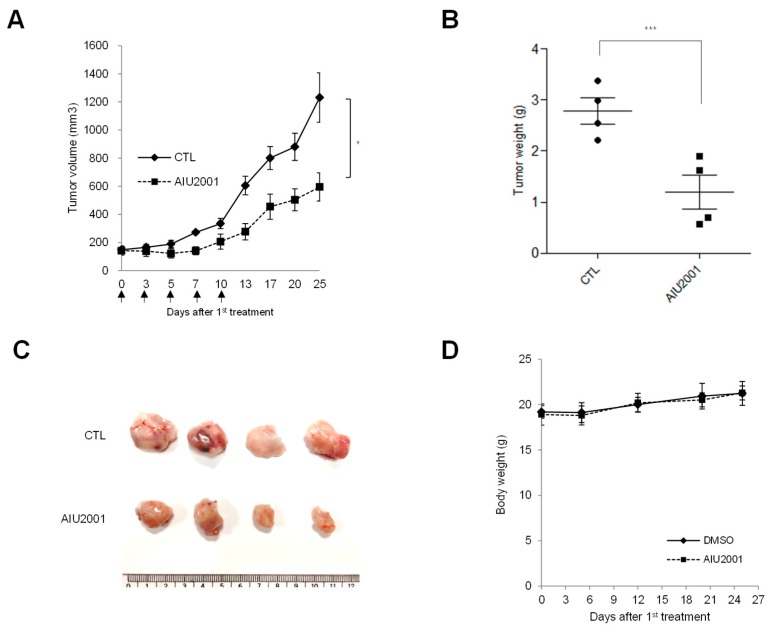
AIU2001 inhibited tumor growth of A549 xenografts in nude mice. (**A**) A549 cells were *s.c.* injected into the thigh of the right hind leg of BALB/c nu/nu mice (*n* = 4/group). Two weeks after tumor cell injection, AIU2001 (20 mg/kg) or DMSO was administered (*i.p.*) once per two or three days for five times in total. The longest (L) and shortest (W) tumor axes were measured, and tumor volume (mm^3^) was calculated as L × W^2^/2. The data shown represent the average tumor volume (* *p* < 0.05). (**B**) The weight of the resected tumors was measured at the end of the experiment (*** *p* < 0.001). (**C**) Image of resected tumors from mice. (**D**) The body weights of A549 tumor xenograft mice were determined twice weekly during the experiments.

We next determined whether the antitumor effect associated with in vitro AIU2001 treatment could be translated into an in vivo xenograft mouse model. Mice were subcutaneously (*s.c.*) implanted with A549 cells in the right hind leg. When the tumors were palpable (average diameter: 150 mm^3^; 14days post-implantation), mice were intraperitoneally (*i.p.*) administered 20 mg/kg AIU2001 or DMSO once per two or three days for five times in total. Compared to the vehicle, the AIU2001 treatment inhibited A549 cell-derived tumor growth by 48.3% (Figure 2A). At necropsy, the average tumor weight of mice treated with AIU2001 or DMSO was 1.2 g or 2.8 g, respectively (Figure 2B,C). In addition, we measured the body weight of mice to determine AIU2001 toxicity and observed that the body weights of control mice and AIU2001-treated mice did not differ (Figure 2D). Thus, AIU2001 exhibited antitumor activity both in vitro and in vivo.

### 2.2. AIU2001 Increased Apoptotic Cell Death in Human NSCLC Cells

**Figure 3:**
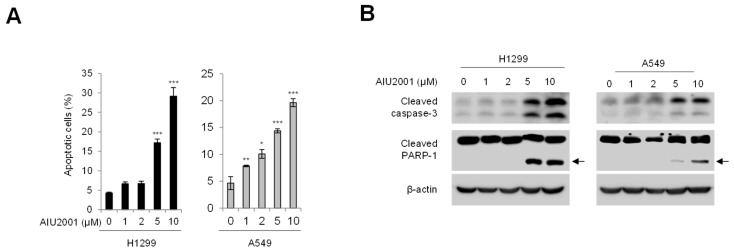
AIU2001 induced apoptotic cell death in NSCLC cells. H1299 and A549 cells were treated with AIU2001 at the indicated concentrations for 48 h. (**A**) The apoptotic cells were determined using APC-conjugated annexin V/PI staining. Cell populations were gated into four groups as described in the Materials and methods. Bar graphs represent the mean percentage of early apoptotic cells (annexin V-positive/PI-negative) and late apoptotic cells (annexin V-positive/PI-positive). Data represent the mean ± SD of three independent experiments. * *p* < 0.05, ** *p* < 0.01, *** *p* < 0.001 versus respective DMSO-treated cells. (**B**) H1299 and A549 cell lysates were subjected to immunoblotting for detection of cleaved caspase-3 and PARP-1. β-actin was used as a loading control.

As AIU2001 inhibited cancer cell viability, we sought to determine whether AIU2001 induced apoptotic cell death in H1299 and A549 cells. The apoptotic cell populations of these cell lines were detected using FACS analysis with annexin V/propidium iodide (PI) staining (Figure 3A). The number of H1299 or A549 cells undergoing both early stage (annexin V-positive/PI-negative) and late-stage (annexin V-positive/PI-positive) apoptosis increased significantly by 6.7- or 4.2-fold, respectively, following treatment with 10 µM of AIU2001. In addition, the AIU2001 treatment increased cleavage of caspase-3 and PARP-1 in both cell lines (Figure 3B). Taken together, these results indicated that AIU2001 induced apoptotic cell death in human NSCLC H1299 and A549 cells.

### 2.3. AIU2001 Induced Cell Cycle Arrest and Suppressed DNA Damage Repair

**Figure 4:**
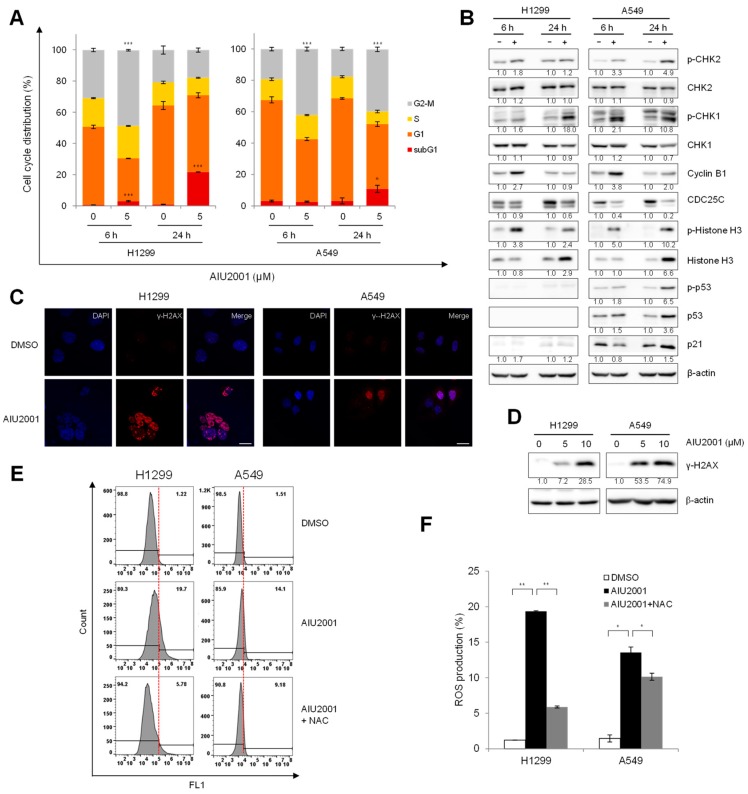
AIU2001 induced cell cycle arrest in G2/M phase and DNA damage in NSCLC cells. (**A**) H1299 and A549 cells were treated with 5 μM AIU2001 for 6 h or 24 h and stained with PI. Cell cycle distribution analyzed using flow cytometry. Data represent mean ± SD of three independent experiments. * *p* < 0.05, *** *p* < 0.001 versus respective DMSO-treated cells. (**B**) Cell lysates were prepared at 6 h or 24 h after treatment with 5 μM AIU2001 to detect the indicated proteins using immunoblotting. (**C**) H1299 and A549 cells were treated with 5 μM AIU2001 and immunostained for γ-H2AX foci (red) and nuclei (DAPI: blue). The photographs were captured at 400× magnification. All bars represent 20 μm. (**D**) The cells were treated with the indicated concentrations of AIU2001 and were immunoblotted for detection of γ-H2AX expression. (**E**,**F**) H1299 and A549 cells were treated with 5 mM NAC for 1 h, followed by AIU2001 for 24 h after treatment with 2′,7′-dichlorodihydrofluorescin diacetate (CM-H_2_DCFHDA) for 30 min. Total cellular ROS production was measured using flow cytometry. Data are representative of three independent experiments (**E**). The bar graph shows the quantitative analysis of FACS data (**F**). Data represent the mean ± standard deviation of three independent experiments. * *p* < 0.05, ** *p* < 0.01 versus the corresponding values.

To determine whether AIU2001 caused cell cycle arrest, we investigated the cell cycle distribution of AIU2001-treated H1299 and A549 cells using flow cytometry analysis. Both cell lines showed a G2/M phase arrest 3 h, 6 h, or 24 h after treatment with AIU2001 (Figure 4A and Supplementary Figure S2). Consistent with the results of Figure 2, we observed a significant increase in the percentage of 24 h AIU2001-treated H1299 (23.1%) and A549 (3.3%) cells in the sub-G1 phase (apoptotic cells) compared to that of the control. To determine the molecular event associated with AIU2001-elicited cell cycle arrest, we determined the expression levels of relevant proteins in the CHK- and p53-dependent pathways in the H1299 and A549 cells arrested in the G2/M phase [18,19,20,21]. AIU2001 treatment increased the phosphorylation of CHK1 at Ser345 and that of CHK2 at Thr68 in both cell lines. Several studies have reported that cyclin B1 level increases in cancer cells arrested in the G2/M phase [22,23,24]. Compared to in DMSO-treated cells, we observed significant increase in cyclin B1 and phosphorylated histone H3 levels and decrease in CDC25C level among the key regulators of the G2 to M phase transition in AIU2001-treated cells. The tumor suppressor p53 is a key checkpoint protein in p53 wild-type cells. It is noteworthy that the expression of phosphorylated p53 and p21 increased in A549 cells harboring p53 wild-type after AIU2001 treatment, but not in p53-deficient H1299.

As treatment with AIU2001 results in accumulation of cells in the G2/M phase, we investigated whether AIU2001-induced cell cycle arrest was due to accumulation of DNA damage. The response of the DNA DSB repair machinery to severe DNA damage was determined by assessing the phosphorylation on Ser139 of H2AX (γ-H2AX), which is an indicator of the presence of DNA DSBs produced under stress conditions, such as oxidative or replication stress. Compared to the corresponding DMSO-treated cells, high levels of γ-H2AX foci were observed in AIU2001-treated H1299 and A549 cells using immunofluorescence confocal microscopy, and similar results were observed regarding the phosphorylation level of H2AX using immunoblot analysis (Figure 4C,D). ROS might induce DNA DSBs in AIU2001-treated cells. Hence, we determined whether AIU2001 *per* se contributed to increase in ROS generation. Flow cytometry analysis showed that treatment of H1299 and A549 cells with 5 μM AIU2001 distinctly increased ROS levels, which was reduced by treatment with NAC, a general free radical scavenger (Figure 4E,F). We postulated that AIU2001-induced ROS may induce severe accumulation of DSBs, which may be involved in the antitumor activity. 

### 2.4. AIU2001 Induced DNA Damage and Suppressed DNA Damage Repair in NSCLC Cells

**Figure 5:**
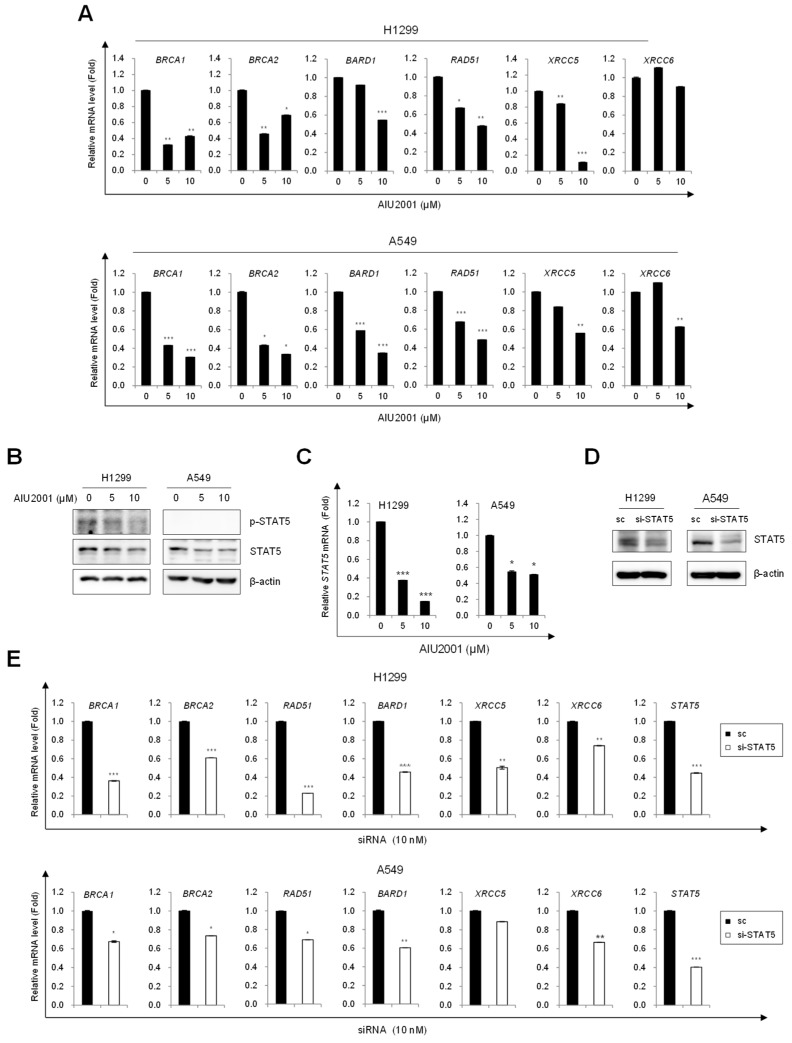
AIU2001 inhibited genes related to DNA damage repair in NSCLC cells. (**A**) H1299 and A549 cells were treated with the indicated concentrations of AIU2001 for 24 h, followed by assessment of the indicated mRNA levels using qPCR. (**B**,**C**) Cell lysates were prepared and analyzed using immunoblotting for phospho-STAT5 and STAT5 (**B**) or using qPCR for *STAT5* mRNA (**C**). (**D**,**E**) H1299 and A549 cells were transfected with either scrambled siRNA (sc) or STAT5 si-RNA (si-STAT5) for 24 h. Expression level of STAT5 was determined using immunoblotting (**D**) and the mRNA levels of the indicated genes were quantified using qPCR (**E**). Data are from one representative experiment which was repeated thrice. * *p* < 0.05, ** *p* < 0.01, *** *p* < 0.001 versus respective vehicle controls.

A recent study demonstrated that quizartinib inhibits the DNA repair system in AML cells harboring the FLT3-ITD mutation [14]. We investigated whether AIU2001 inhibited DNA repair in NSCLC cells. Consistent with the results obtained using FLT3-ITD mutant AML cells, AIU2001 treatment reduced the mRNA levels of the HR (*BRCA1*, *BRCA2*, *BARD1*, and *RAD51*) and NHEJ (*XRCC5* and *XRCC6*) machineries in A549 and H1299 cells (Figure 5A). 

STAT5 is a downstream effector of FLT3 signaling in FLT3(ITD)-positive AML cells and we observed that AIU2001 dose-dependently inhibited phosphorylated STAT5 in Molm-13 and MV4-11 cells (Supplementary Figure S3). However, STAT5 is expressed at low level and is not considered an oncogenic molecule in NSCLC cells. We could detect that AIU2001 inhibited phosphorylated STAT5 in H1299 cells, but we did not detect it in A549 cells using immunoblotting (Figure 5B). The expression level of the *STAT5* mRNA was determined instead of the protein and it was downregulated by AIU2001 treatment in H1299 and A549 cells (Figure 5C). Consistent with the results of AIU2001, quizartinib decreased the expression of *STAT5* mRNA and DNA damage repair genes in both cells (Supplementary Figure S4). We speculated that STAT5 inhibition may downregulate the DNA repair genes. Interestingly, siRNA-mediated *STAT5* knockdown inhibited HR and NHEJ genes in both cell lines (Figure 5E). These results indicated that AIU2001 downregulated the DNA damage repair genes and regulated the cell cycle-related molecules, leading to G2/M phase arrest, which may contribute to cell death in solid tumors.

### 2.5. Combination of AIU2001 and a PARP Inhibitor or IR Synergistically Inhibited the Growth of H1299 and A549 Cells

**Figure 6:**
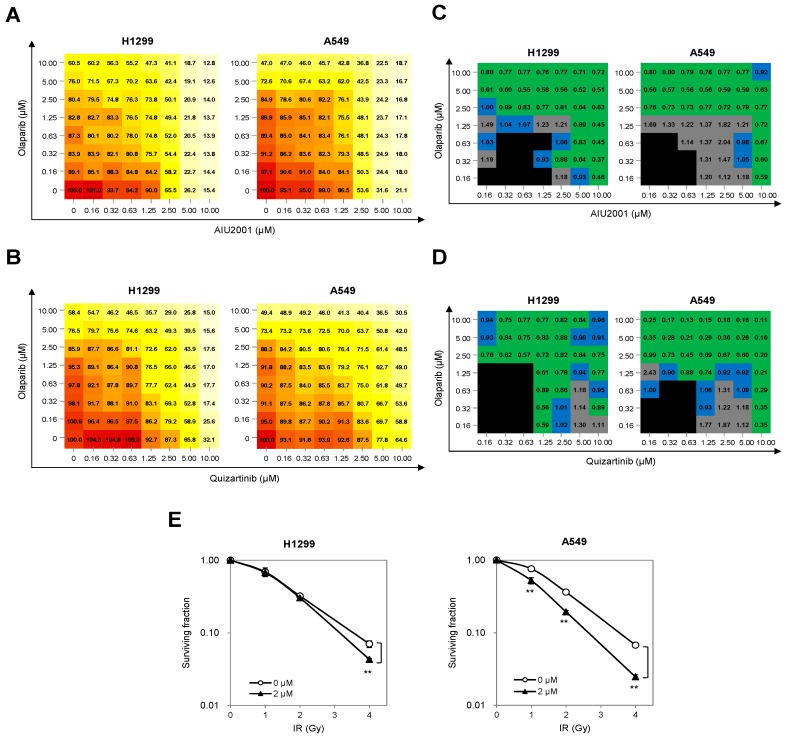
Combination of AIU2001 and olaparib or IR synergistically inhibited cell growth in H1299 and A549 cells. (**A,B**) H1299 and A549 cells subjected to monotherapy or combination therapy with AIU2001 (**A**) and quizartinib (**B**) or olaparib at the indicated concentrations. Cell viability was determined using the MTT assay after five days of treatment. Relative viability (normalized to DMSO-treated cells) is shown for each combination at the indicated concentrations. The data are from one representative data, which was repeated thrice. (**C**,**D**) Summary of table shows CI scores of AIU2001 and olaparib combined at the indicated concentrations in H1299 (**C**) and A549 cells (**D**). CI scores were calculated using the Compusyn software and categorized as synergistic (CI < 0.9, green), additive (1.1 ≥ CI ≥ 0.9, blue) or antagonistic (CI > 1.1, gray). The not significant ones are marked in black. Each CI score represents data derived from the indicated concentrations of single- and paired compound treatments with more than three independent experiments. (**E**) A549 and H1299 cells were treated with AIU2001 for 2 h before IR and then incubated for 10 days. Colonies with more than 50 cells were counted. ** *p* < 0.01 versus respective vehicle controls.

As AIU2001-mediated HR and NHEJ downregulation mimicked the “BRCAness/DNA-PKness” phenotype and induced DSBs, we investigated whether AIU2001 was sensitive to cellular synthetic lethality exerted by a PARP-1 inhibitor. Olaparib is the first PARP1 and PARP2 inhibitor approved for treatment of refractory ovarian cancer harboring BRCA1 or BRCA2 mutation. As a proof of concept, H1299 and A549 cells were treated with the indicated concentrations of AIU2001 and 0.16 to 10 µM olaparib, and cell viability was assessed using MTT to evaluate the synergistic effect of these two compounds. Compared to treatment with AIU2001 or olaparib alone, the combination treatment showed strong synergistic effect in H1299 and A549 cells, decreasing cell viability in a dose-dependent manner (Figure 6A). To interpret the effects of the drug combination, cell viability observed with each concentration pair of AIU2001-olaparib was converted to a CI score using the CompuSyn software by Chou-Talalay [25]. The CI scores were categorized as synergistic (CI < 0.9, green), additive (1.1 ≥ CI ≥ 0.9, blue), or antagonistic (CI > 1.1, gray), with black indicating no significance. We observed synergistic growth inhibition with a wide range of concentrations of AIU2001 and olaparib in both cell lines (Figure 6B). To determine whether quizartinib also exhibited synergistic growth inhibition when H1299 and A549 cells were treated with olaparib, MTT cell viability assay was performed in the same manner as with AIU2001 and olaparib. As expected, the quizartinib-olaparib combination showed a dramatic synergistic effect on growth retardation, and most CI scores of each drug-pair had a synergistic effect (Figure 6C,D). As radiotherapy exerts enhanced antitumor effect when the DNA repair system is suppressed, we investigated whether AIU2001 enhanced the radiosensitivity of H1299 and A549 cells. The clonogenic survival assay revealed that AIU2001 radiosensitized both cell lines. Dose enhancement ratios (DER) of AIU2001 with 2 μM at surviving fraction of 0.1 over DMSO-treated cells were 1.15 and 1.33 in H1299 and A549 cells, respectively. Taken together, these results support the conclusion that AIU2001 suppressed the expression of the DNA repair genes and enhanced the sensitivity of NSCLC cells to a potent PARP inhibitor and IR.

## 3. Discussion

In this study, we screened novel kinase inhibitors targeting class III RTK activity from synthesized in-house compounds using in vitro kinase profiling and cell viability assays in various cancer cell lines. Among the compounds, AIU2001 was identified as a potent class III RTK inhibitor with anti-cancer effects against solid cancer cell lines, including NSCLC cells, as well as AML cells. We observed that AIU2001 inhibited DNA damage repair and induced ROS production. Subsequent experiments demonstrated that AIU2001 induced the “BRCAness/DNA-PKness” phenotype, which contributed to the cytotoxicity of NSCLC cells and enhanced the sensitivity to a PARP inhibitor. 

As quizartinib is a more potent and selective second-generation FLT3 inhibitor than the first-generation FLT3 inhibitor midostaurin, which was approved for AML treatment in 2017, it can inhibit multiple class III RTKs, including the structurally similar FLT3 and c-KIT. AIU2001 also exhibited a pan-class III RTK inhibitory effect. 

Gain-of-function mutations of FLT3 and KIT play critical roles in the oncogenesis of AML [26,27,28]. In particular, the most prevalent genetic mutations associated with AML have been detected in FLT3. Unlike other AML treatments, quizartinib has not been studied extensively due to its limited efficacy on solid tumors, which harbor few genetic mutations in FLT3, with the exception of gastrointestinal stromal tumors (GISTs), which harbor gain-of-function mutations in the KIT receptor. Most FLT3 inhibitors possess antitumor activity as they can block the activated STAT5 pathway in AML cells with FLT3-ITD, and combination approaches with quizartinib or midostaurin and other anti-cancer drugs were mainly studied in myeloid leukemia and not solid tumors [29]. Recent studies have shown that inhibition of FLT3(ITD) activity by quizartinib downregulated the DNA repair proteins and indicated that quizartinib sensitized FLT3(TID)-positive AML cells to synthetic lethality triggered by PARP inhibitors via inhibition of the DSB repair pathways. This is because only FLT3(ITD)-positive leukemia cells, but not wild-type FLT3 AML cells, accumulate ROS-induced DSBs, but can survive due to the enhanced DNA repair activities [14,30]. According to Maifrede et al., the quizartinib-mediated inhibition of DNA repair might be because of inhibition of the signaling pathway of class III RTK [14]. Our data also demonstrated that STAT5 knockdown significantly inhibited the DNA repair genes in H1299 and A549 cells.

Class III RKT inhibitors are not considered for NSCLC therapy as FLT3 and c-KIT are not oncogenic drivers of NSCLC. Furthermore, the cellular function of STAT5 signaling has not been extensively studied in NSCLC cells. However, we found common features between FLT3(ITD)-positive AML cells and NSCLC cells, including strong resistance to DNA-damaging therapeutic agents due to high DNA repair system compared to wild-type FLT3 cells or normal cells. Thus, the question arises as to whether a FLT3 inhibitor could be effective on NSCLC cells when DNA repair genes are essential for the survival of NSCLC cells, even those with wild-type FLT3 [14,30,31,32]. A large number of genetic alterations (> 200) have been identified in human NSCLC; for example, the v-Ki-ras2 Kirsten rat sarcoma virus oncogene (KRAS) and epidermal growth factor receptor (EGFR) are the most commonly mutated oncogenes that drive the pathogenesis of lung cancer [33,34,35]. Amplification of KRAS or EGFR signaling is associated with DNA damage repair. Indeed, activation of mutated KRAS cancer cells is highly dependent on RAD51 for survival, KRAS mutation-dependent AKT1 stimulates HR and NHEJ activities for DNA DSB repair, and nuclear translocation of EGFR is associated with DNA-PKs for DSB repair [36,37,38]. Several studies have demonstrated that combination treatment with inhibitors targeting HR and/or NHEJ with IR or cytotoxic drugs sensitizes NSCLC cells [39,40]. We showed that AIU2001 inhibited cell viability, which is probably due to accumulated ROS-mediated DNA damage and suppression of DNA repair via downregulation of STAT5. Subsequently, AIU2001 treatment induced cell cycle arrest at the G2/M phase with accumulation of a significant sub-G1 population in H1299 and A549 cells. The mechanisms responsible for AIU2001-mediated downregulation of HR and NHEJ and the relationship between STAT5 and DNA damage repair genes have not been completely uncovered in this study, which is a limitation of this study. Further studies are required to investigate the mechanisms associated with AIU2001-induced downregulation of DNA damage repair genes.

As one of the clinical applications of synthetic lethal treatment, PARP inhibition has shown promising effect in the treatment of patients with tumors harboring mutations in BRCA1 or BRCA2. Recently, combination therapies with an FDA-approved PARP inhibitor and DNA damaging agents have been studied in NSCLC to expand the clinical indications of FDA-approved PARP inhibitors [41]. AIU2001 downregulated the expression of HR and NHEJ genes, resulting in “BRCAness” and “DNA-PKness” phenotype, which contributed to synthetic lethality with PARP inhibition or IR in NSCLC cells. Our results demonstrated that the combination of AIU2001 and olaparib or IR significantly inhibited cancer cell growth. It is noteworthy that FLT3 inhibitors can be considered partner drugs of PARP inhibitors or radiotherapy for inducing synthetic lethality in solid tumors.

In summary, our study demonstrated that AIU2001 exhibited a potent pan-class III RKT inhibitor activity and cytotoxicity toward NSCLC cells. AIU2001 treatment suppressed DNA repair genes and induced DNA damage, resulting in induction of cell cycle arrest at the G2/M phase and apoptotic cell death. We have also shown that the combination of AIU2001 with the PARP inhibitor olaparib or IR considerably delayed cell proliferation. In this context, AIU2001 may act as an effective anti-cancer therapeutic for solid tumors as well as for AML although further investigations are warranted. 

## 4. Materials and Methods

### 4.1. Chemical Synthesis

All chemical reagents were commercially available and were used without further purification. Melting points were determined using a Kruess M5000 melting point apparatus and were not corrected. Proton NMR spectra were recorded on an Avance-500 (Bruker, Billerica, MA, USA) at 500 MHz. Chemical shifts were reported in ppm units with Me4Si as the reference standard. Mass spectra were recorded on a JEOL, JMS-600W VG Trio-2 GC–MS. Reaction products were purified using flash column chromatography with silica gel 60 (230–400 mesh, Merck, Mumbai, India) and monitored using TLC on precoated silica gel 60 F254 (Merck). Spots were visualized under UV light (254 nm) after staining with phosphomolybdic acid (PMA) or Hanessian’s solution. The details have been described in the supplementary materials and methods section and Supplementary Scheme S1. 

### 4.2. Prediction of Drug-Protein Interactions

For the docking study, the targets were prepared and minimized using the Cresset Flare software [42], the grid box was defined according to the clustered ligand of downloaded FLT3 and c-KIT, and the docking calculations were performed using the Cresset Flare software in normal mode and default settings.

### 4.3. Reagents

MTT was purchased from Amresco (Solon, OH, USA). The primary antibodies used in this study included the following; anti-PARP1, anti-cleaved caspase 3, anti-phospho-CHK1, anti-CHK1, anti-phospho-CHK2, anti-CHK2, anti-phospo-p53, anti-p53 (Cell Signaling Technology; Danvers, MA, USA), anti-Cyclin B1, anti-CDC25C, anti-phospho-histone H2AX (Santa Cruz Biotechnology; Dakkas, TX, USA), and anti-β-actin (Sigma-Aldrich; St. Louis, MO, USA) antibodies. Olaparib (AZD2281) and quizartinib (AC220) were purchased from Selleckchem (Houston, TX, USA) and dissolved in DMSO (Sigma Aldrich). N-acetyl-L-cysteine (NAC) was purchased from Sigma Aldrich. CM-H_2_DCFDA was purchased from Invitrogen (Carlsbad, CA, USA).

### 4.4. Cell Culture

H1299, A549, and H460 human lung cancer cell lines and BEAS2B and CCD18-Lu human normal lung cells (American Type Culture Collection, Manassas, VA, USA) were maintained in Roswell Park Memorial Institute (RPMI) 1640 medium (H1299, A549, and H460; Welgene, Gyeonsangbukdo, Korea), Dulbecco’s modified Eagles medium (DMEM; BEAS2B; Welgene) or Eagle’s minimum essential medium (EMEM; CCD18-Lu; Welgene) supplemented with 10% fetal bovine serum (FBS; Welgene) and 100 units/mL penicillin streptomycin solution (Gibco, Grand Island, NY, USA) at 37 °C in a humidified 5% CO_2_ atmosphere.

### 4.5. Cell Viability Assay

Cell viability was assessed using the MTT colorimetric assay. Cells (1 × 10^3^ cells/well) were seeded into 96-well plates and treated with the various concentrations of each compound or combination of two compounds. After five days of treatment, 10 μL MTT (0.5 mg/mL) was added, and further incubated for 3 h. After removal of the supernatant, the resultant pellet was dissolved in DMSO. The absorbance of the resultant formazan was measured at 540 nm using a plate reader (Multiskan EX; ThermoLabsystems, Waltham, MA, USA).

### 4.6. In Vitro Kinase Assay

Initial kinase profiling of AIU2001 against a panel of 53 functional kinases was performed by Eurofins (Eurofins Pharma Discovery, UK) and the IC50 values of the kinases were determined by Reaction Biology Corp. (Malvern, PA, USA).

### 4.7. Immunoblot Analysis

Cell lysates were prepared by extracting proteins with TNN buffer (40 mM Tris-Cl pH 8.0, 0.2% NP-40, 120 mM NaCl) or radioimmunoprecipitation assay (RIPA) lysis buffer (Millipore, Billerica MA, USA) supplemented with a protease inhibitor cocktail (Thermo Fisher Scientific, Rockford, IL, USA). Equal amounts of proteins were separated using SDS-PAGE on 8–13% gels, and transferred to nitrocellulose membranes (Bio-Rad, Hercules, CA, USA). The membranes were blocked with 5% skim milk in Tris-buffered saline-Tween 20 (TBST) (150 mM NaCl, 10 mM Tris, 0.2% Tween20), followed by overnight incubation with primary antibodies at 4 °C. The blots were developed using peroxidase-conjugated secondary antibody and the immunoreactive proteins were visualized using enhanced chemiluminescence (ECL) reagents, according to the manufacturer’s recommendations (Amersham, GE Healthcare, Buckingamshire, UK). The protein bands were visualized using a digital imaging system (ImageQuant LAS 4000 mini; GE Healthcare, UK). The protein levels were analyzed using Image J software (National Institutes of Health, Bethesda, MD, USA). Experiments were repeated at least thrice.

### 4.8. Annexin V/PI-Based Flow Cytometric Analysis

Annexin V assays were performed according to the manufacturer’s protocol (BD Pharmingen, San Diego, CA, USA). Briefly, 10,000 cells were plated into 60-mm plates and treated with varying concentrations of AIU2001 for 48 h. The cells were harvested and incubated with 4 μL allophycocyanin (APC)-conjugated annexin V (20 μg/mL) and 4 μL PI (50 μg/mL) for 15 min. Fluorescence analyses were performed using flow cytometry (CyFlow Cube 6; Sysmexpartec, Goerlitz, Germany). Cells were classified as early apoptotic (annexin V-positive/PI-negative), late apoptotic/necrotic (annexin V-positive/PI-positive), necrotic/dead (annexin V-negative/PI-positive), and live (annexin V-negative/PI-negative). Flow cytometry data was analyzed using FlowJo software (TreeStar Inc., Ashland, OR, USA).

### 4.9. Cell Cycle Analysis

Samples were collected at the indicated time points and fixed in 70% cold ethanol overnight. For cell cycle analysis, the fixed cells were treated with RNase for 20 min before addition of 50 μg/mL PI and analyzed using FACS Calibur™ (BD Biosciences, San Jose, CA, USA).

### 4.10. Tumor Xenograft Mouse Models

A549 human lung cancer cell xenografts were initially established by *s.c.* implanting 1 × 10^6^ cultured cells into the thigh of the right hind leg of six week old mice. When tumor volumes had reached approximately 150 mm^3^, AIU2001 (10 mg/kg) was administered *i.p.* once per two or three days for five times in total. All animal experiments were reviewed and approved by the Institutional Animal Care & Use Committee of Korea Institute of Radiological and Medical Sciences (kirams2018-0063, 6 December 2018). 

### 4.11. Tumor Measurement

Two axes of the tumor (L, longest axis; W, shortest axis) were measured twice per week after irradiation using Vernier calipers. Tumor volume was calculated as (L × W^2^)/2 (mm^3^).

### 4.12. RNA Extraction and Quantitative Polymerase Chain Reaction (qPCR) Analysis

RNA was extracted using TRIzol^®^ RNA isolation reagent (ThermoFisher Scientific). RNA was reverse-transcribed into cDNA using the M-MLV reverse transcriptase (Enzynomics, Daejeon, Korea) and RNase inhibitor (Promega, Madison, WI, USA). The sequence of the primers targeting human *BRCA1*, *BRCA2*, *RAD51*, *BARD1*, *XRCC6*, and *XRCC5* are as follows: *BRCA1* sense 5′-CTGAAGACTGCTCAGGGCTATC-3′, *BRCA1* antisense 5′-AGGGTAGCTGTTAGAAGGCTGG-3′, *BRCA2* sense 5′-GGCTTCAAAAAGCACTCCAGATG-3′, *BRCA2* antisense 5′-GGATTCTGTATCTCTTGACGTTCC-3′, *RAD51* sense 5′-CTCAGCCTCCCGAGTAGTTG-3′, *RAD51* antisense 5′-CATCACTGCCAGAGAGACCA-3′, *BARD1* sense 5′-GCCAAAGCTGTTTGATGGAT-3′, *BARD1* antisense 5′-CGAACCCTCTCTGGGTGATA-3′, *XRCC6* sense 5′-AAAAGACTGGGCTCCTTGGT-3′, *XRCC6* antisense 5′-TGTGGGTCTTCAGCTCCTCT-3′, *XRCC5* sense 5′-CGACAGGTGTTTGCTGAGAA-3′,and *XRCC5* antisense 5′-TCACATCCATGCTCACGATT-3′. *β-actin* was used as a housekeeping gene for normalization. The cDNA was quantified using real-time PCR with SYBR Green/fluorescein qPCR master mix (ThermoScientific, Carlsbad, CA, USA) on a Lightcycler 96 system (Roche Diagnostics, Mannheim, Germany) according to the manufacturer’s protocol.

### 4.13. Immunofluorescence and Foci Assay

For γ-H2AX and 4′,6-diamidino-2-phenylindole (DAPI) staining, cells were fixed in 4% paraformaldehyde for 15 min at room temperature and permeabilized with 0.1% Triton X-100 in PBS for 20 min. The cells were then incubated with 1:200 dilution of anti-phosho-H2AX (Ser139) antibody overnight at 4 °C. AlexaFluor 594-conjugated anti-mouse IgG antibody (Abcam, Cambridge, UK) was used at 1:400 dilution for 1 h at room temperature. The slides were mounted in mounting medium (DAKO, Santa Clara, CA, USA) with DAPI (ThermoFisher Scientific) before imaging. Images were acquired using an LSM880 laser scanning microscope (ZEISS, Jena, Germany). Fluorescent images were captured using the appropriate filters. Images were analyzed using the Image J and ZEN software.

### 4.14. Detection of Intracellular Reactive Oxygen Species (ROS)

DMSO- or AIU2001-treated cells (5 × 10^5^) were further treated with 10 μM CM-H_2_DCFH-DA for 30min and then washed with PBS before trypsinization. After detaching with trypsin, the cells were collected, washed, and resuspended in PBS. ROS inhibition was evaluated by treating cells with 5 mM NAC 2 h prior to AIU2001 treatment. Intracellular ROS levels were detected using a flow cytometer (CyFlow cube 6) at excitation/emission wavelengths of 488/525 nm.

### 4.15. Combination Index (CI)

CI scores were calculated using the CompuSyn software by Chou (CompuSyn Inc., Paramus, NJ) [25] based on cell viability after treatment with single- and paired-drug concentrations. The CI equation for two drugs was used:

$$\mathrm{CI}=\frac{\left(D\right)A}{\left(\mathrm{Dx}\right)A}+\frac{\left(D\right)B}{\left(\mathrm{Dx}\right)B}$$

where (Dx)A is the concentration of drug A alone that inhibits *x*%, (Dx)B is the concentration of drug B alone that inhibits *x*%, (D)A or (D)B is the portion of drug A or drug B in the combination (D)A + (D)B that inhibits *x*%. Thus (D)A + (D)B also inhibits *x*%.

### 4.16. Clonogenic Assay

Cells were seeded on 60-mm culture dishes at various densities and then treated with DMSO or 2 μM AIU2001. After 2 h, the cells were treated with the indicated doses of ^137^Cs γ-radiation. After 10 days, the colonies were fixed and stained with 1.5% methylene blue (Sigma Aldrich) in methanol solution. Colonies containing > 50 cells were counted. The dose enhancement ratio (DER) was calculated as the dose (Gy) of radiation that yielded a surviving fraction of 0.1 for DMSO-treated cells divided by that dose for AIU2001-treated cells. The experiment was performed in triplicate.

### 4.17. Statistical Analysis

Results are shown as means ± the standard deviations (SDs). Data were analyzed using two-tailed Student’s t-tests. Analysis of variance (ANOVA) and Tukey’s post hoc test were used for the two or three-group comparisons. Differences between groups with *p*-values < 0.05 were considered statistically significant.

## Figures and Tables

**Table 1:** In vitro kinase inhibition profile of AIU2001.

Kinase	IC_50_ (nM)	Inhibitory Activity at 10 μM (%)
FLT3	206.4	92
FLT3 (D835Y)	108.1	75
FLT3 (ITD)	63.2	98
FLT4	785.5	81
c-KIT	223.3	88
c-KIT (V560G)	53.4	91

## Supplementary Materials

Supplementary materials can be found at https://www.mdpi.com/1422-0067/20/19/4728/s1.
