# Supplementary material for: Antitumor Activity of a Novel Tyrosine Kinase Inhibitor AIU2001 Due to Abrogation of the DNA Damage Repair in Non-Small Cell Lung Cancer Cells
Source: Int J Mol Sci. 2019 Sep 24;20(19):4728. doi: 10.3390/ijms20194728 (PMC6801739; doi:10.3390/ijms20194728)
Supplement: Supplementary file 1 [file ijms-20-04728-s001.pdf]

Supplementary Materials

Supplementary Scheme 1.

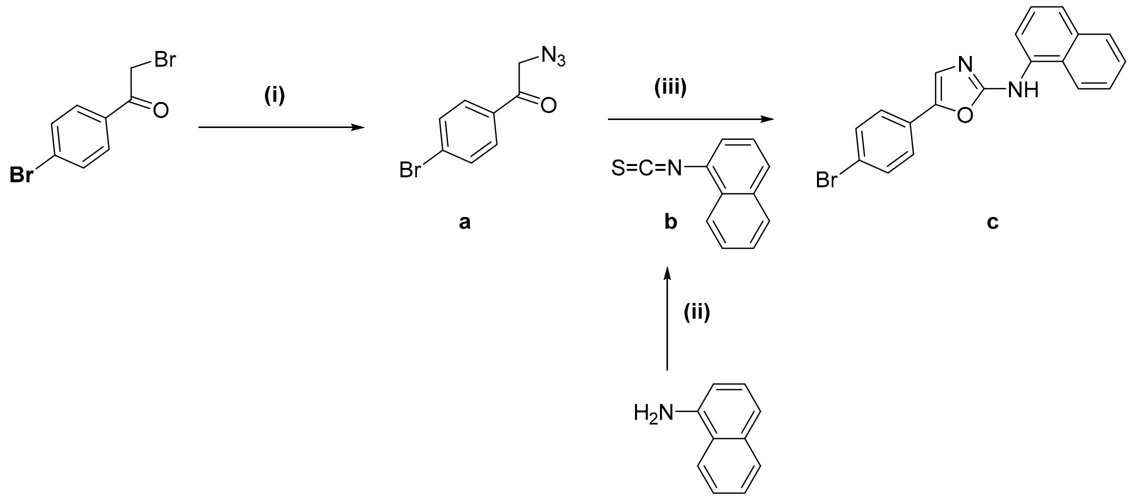

Reagents and conditions: (i) Sodium azide, acetone for 16 h; (ii) 1-naphthylamine, thiophosgene, DCM for 4 h; (iii) PPh3, dioxane 90°C for 4 h.

Supplementary Table 1. Kinase inhibitory activities of AIU2001 (10 μM) *in vitro*

**Kinase**	**Inhibitory activity (%)**
Abl	51
ALK	0
AMPKα1	25
ARK5	75
Aurora-A	28
B-RAF	0
CDK1/cyclinB	38
CDK2/cyclinA	39
CDK5/p35	58
CHK1	0
CHK2	14
CK1γ1	37
CK2	10
c-KIT	88
c-KIT (D816H)	48
c-KIT (V560G)	91
cSRC	22
DAPK1	12
DDR2	55
EGFR	10
EGFR(T790M,L858R)	4
EPHB4	10
ERBB2	0
ERBB4	1
FAK	16
FGFR1	1
FGFR2	1
FLT1	44
FLT3	92
FLT3 (ITD)	98
FLT3(D835Y)	75
FLT4	81
HIPK2	0
IGF-1R	0
IKKα	7
MAPK2	19
MEK1	0
Met	1
p70S6K	8
PDGFRα	48
PDGFRα(D842V)	23
PDK1	19
PIM-1	37
PKBα	24
PKCα	9
ROCK-I	0
ROS	2
SRC (1-530)	10
TAK1	15
TGFBR1	12
TrkA	40
WEE1	8
ZAP-70	0
PI3 Kinase (p110a/p85a)	7

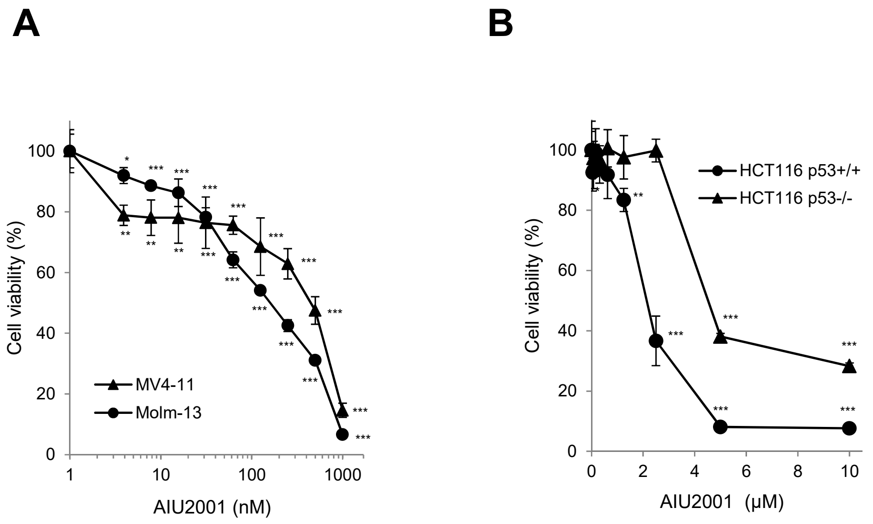

Supplementary Figure 1. AIU2001 inhibited the proliferation of various cancer cells. (A and B) Human AML cells (Molm-13 and MV4-11; A) and human colorectal cancer (CRC) cells (HCT116/p53$^{+/+}$ and HCT116/p53$^{-/-}$; B) were treated with AIU2001 at the indicated concentrations for 5 days and viable cells were evaluated using the CCK-8 assay for AML cells and the MTT assay for CRC cells. **p* < 0.05, ***p* < 0.01, ****p* < 0.001 versus respective vehicle controls.

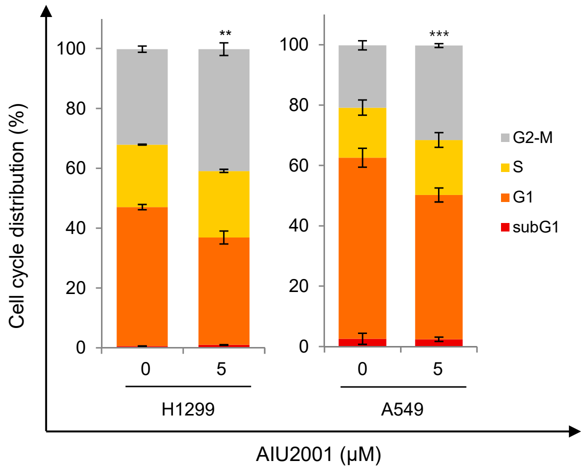

Supplementary Figure 2. AIU2001 induced cell cycle arrest in the G2/M phase. H1299 and A549 cells were treated with 5 μM AIU2001 for 3 h and stained with PI. The cell cycle distribution was analyzed using flow cytometry. Data represent mean ± SD of three independent experiments. ***p* < 0.01, ****p* < 0.001 versus respective vehicle controls.

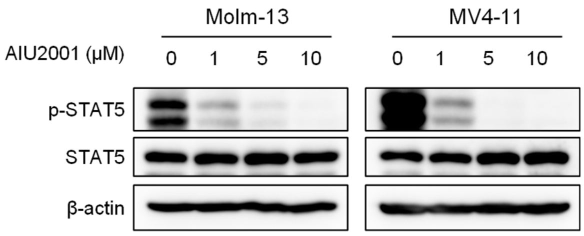

Supplementary Figure 3. AIU2001 inhibited downstream effector STAT5 of FLT3 signaling in FLT3(ITD)-AML cells. Molm-13 and MV4-11 cells were treated with the indicated concentrations of AIU2001 for 1h. Cell lysates were prepared and analyzed using immunoblotting for detecting phospho-STAT5 and STAT5.

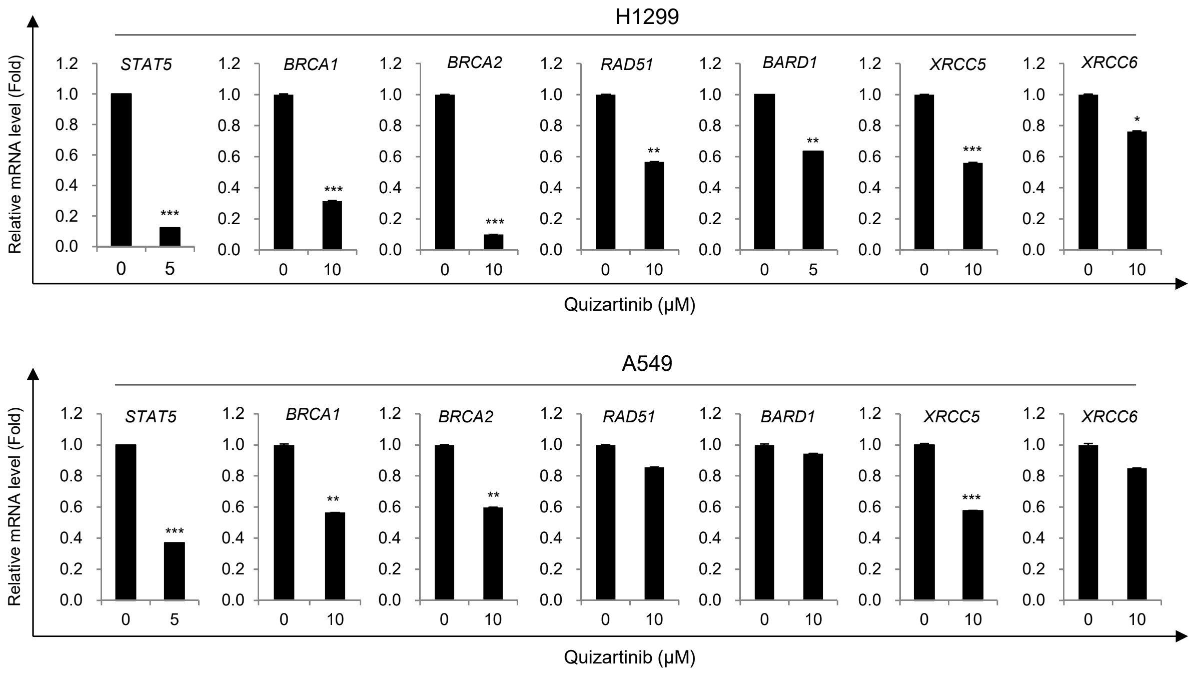

Supplementary Figure 4. Quizartinib inhibited genes related to DNA damage repair in NSCLC cells. H1299 and A549 cells were treated with 10 μM quizartinib for 24 h, followed by assessment of the indicated mRNA levels using qPCR. Data are from one representative experiment, which was repeated thrice. *$p < 0.05$, **$p < 0.01$, ***$p < 0.001$ versus respective vehicle controls.

Supplementary Materials and Methods

Chemical synthesis. General procedure of 5-(4-bromophenyl)-N-(naphthalen-1-yl)oxazol-2-amine synthesis. Azide formation: **(a)** Sodium azide was added to 4′-bromophenacyl bromide (eq.1.0) in acetone (0.2 M) (eq.3.0). After stirring for 16 h at room temperature,the mixture was filtered and concentrated in vacuo to obtain the product (93% yield). Isothiocyanateformation: **(b)** 1-Naphthylamine (eq.1.0) in DCM (0.3 M) was treated with thiophosgene (eq.1.2) and stirred for 4 h. After the reaction was complete, the mixture was diluted with a saturated solution of K2CO3and extracted several times with DCM. The combined organic layers were dried over anhydrous MgSO4, filtered, and concentrated in vacuo. Isoxazole formation: **(c)** PPh3 (eq. 1.0) was added to a stirred solution of azide compounds (a) (eq.1.0) and isothiocyanate compounds (b) (eq.1.0) in dioxane (0.2 M). The mixture was then allowed to heat up to 90°C and stirred for 4h. After the reaction was complete, the solvent was cooled to room temperature and removed under reduced pressure, and the residue was purified using flash column chromatography over silica gel with EtOAc/hexane (1:10) as the eluent to obtain the product (32 % yield).

5-(4-bromophenyl)-N-(naphthalen-1-yl)oxazol-2-amine: **(c)** Yellow solid, m. p. = 183−185 °C, ^{1}H NMR (300 MHz, DMSO-d6) δ 10.19 (s, 1H), 8.30-8.34 (m, 1H), 8.10-8.12 (d, J=7.32 Hz, 1H), 7.92-7.95 (m, 1H), 7.64-7.67 (m,3H), 7.48-7.56 (m, 6H); MS (FAB) m/z 365 (M+).

Cell cultures. Human AML MV4-11 cells were obtained from ATCC. Molm-13 cells were kindly provided by Dr. In-Ki Kim (Asan Medical Center, Seoul, Korea), and $p53^{+/+}$ HCT116 and $p53^{-/-}$ HCT116 cells were kindly provided by Dr. Bert Vogelstein (Johns Hopkins University, Baltimore, MD, USA). The cells were maintained in Iscove’s modified Dulbecco’s medium (IMDM, Gibco; MV4-11) and RPMI (Molm-13, $p53^{+/+}$ HCT116 and $p53^{-/-}$ HCT116) supplemented with 10% FBS (WELGENE) and 100 units/ml penicillin streptomycin solution (GIBCO) at 37°C in a humidified 5% CO_2 atmosphere.

Cell viability. Cytotoxicity assay of AML cells was performed using cell counting kit-8 (CCD-8; Dojindo, Japan) according to the manufacturer's procedure. Briefly, MV4-11 or Molm-13 cells were plated in 96-well plates at a density of 2×10^3 or 1×10^3 cells/well in triplicate, treated with AIU2001 or DMSO, and then incubated for 5 days. After adding the CCK-8 solution (10 μl) to each well and incubating the plates for 3 h, absorbance at 450 nm was measured using a microplate reader (ThermoLabsystems).
